# How Neurobiology Elucidates the Role of Emotions in Financial Decision-Making

**DOI:** 10.3389/fpsyg.2021.697375

**Published:** 2021-07-19

**Authors:** Peter Bossaerts

**Affiliations:** ^1^Faculty of Business and Economics, University of Melbourne, Parkville, VIC, Australia; ^2^Faculty of Economics, University of Cambridge, Cambridge, United Kingdom; ^3^Geneva School of Economics and Management, University of Geneva, Geneva, Switzerland

**Keywords:** emotions, financial decisions and choices, choice theory, neurobiology, neurofinance, decision neuroscience, biomarkers

## Abstract

Over the last 15 years, a revolution has been taking place in neuroscience, whereby models and methods of economics have led to deeper insights into the neurobiological foundations of human decision-making. These have revealed a number of widespread mis-conceptions, among others, about the role of emotions. Furthermore, the findings suggest that a purely behavior-based approach to studying decisions may miss crucial features of human choice long appreciated in biology, such as Pavlovian approach. The findings could help economists formalize elusive concepts such as intuition, as I show here for financial “trading intuition.”

## Highlights

- Neurobiology provides non-behavioral evidence for traditional theories of choice, not only rationalizing them, but also potentially enhancing their out-of-sample predictive power.
- Neurobiology clarifies the true links between economic concepts (e.g., risk aversion), psychological concepts (e.g., feelings), biological ideas (e.g., emotions, genotype) and medical phenomena (e.g., neurological and psychiatric illnesses).
- Neurobiology helps to make sense of elusive concepts such as “gut feeling” or “financial intuition.”
- Emotions are already partially embedded in some of the mathematics of neoclassical choice theory because affection is an integral part of cognition: we decide not only with our brain, but also with our body.
- Neurobiology identifies, from biomarkers, aspects of choice that have been overlooked in traditional behavioral research (e.g., Pavlovian approach-avoidance reflexes).

## Introduction

Decision scientists make sense of observed behavior using “as if” models. In economics, agents choose “as if” optimizing; in psychology, a person avoids gambles “as if” losses loomed larger than gains. Here, we will argue that neurobiology allows decisions scientists to go beyond “as if” modeling. This helps explain, among others, behavioral heterogeneity (why is it that some people are more susceptible to loss/gain framing than others?), and to identify aspect of behavior that have been overlooked in a behavioralist approach (such as Pavlovian approach-avoidance behavior, which is important to understand the genetics behind risk attitudes).

**Figure 1:**
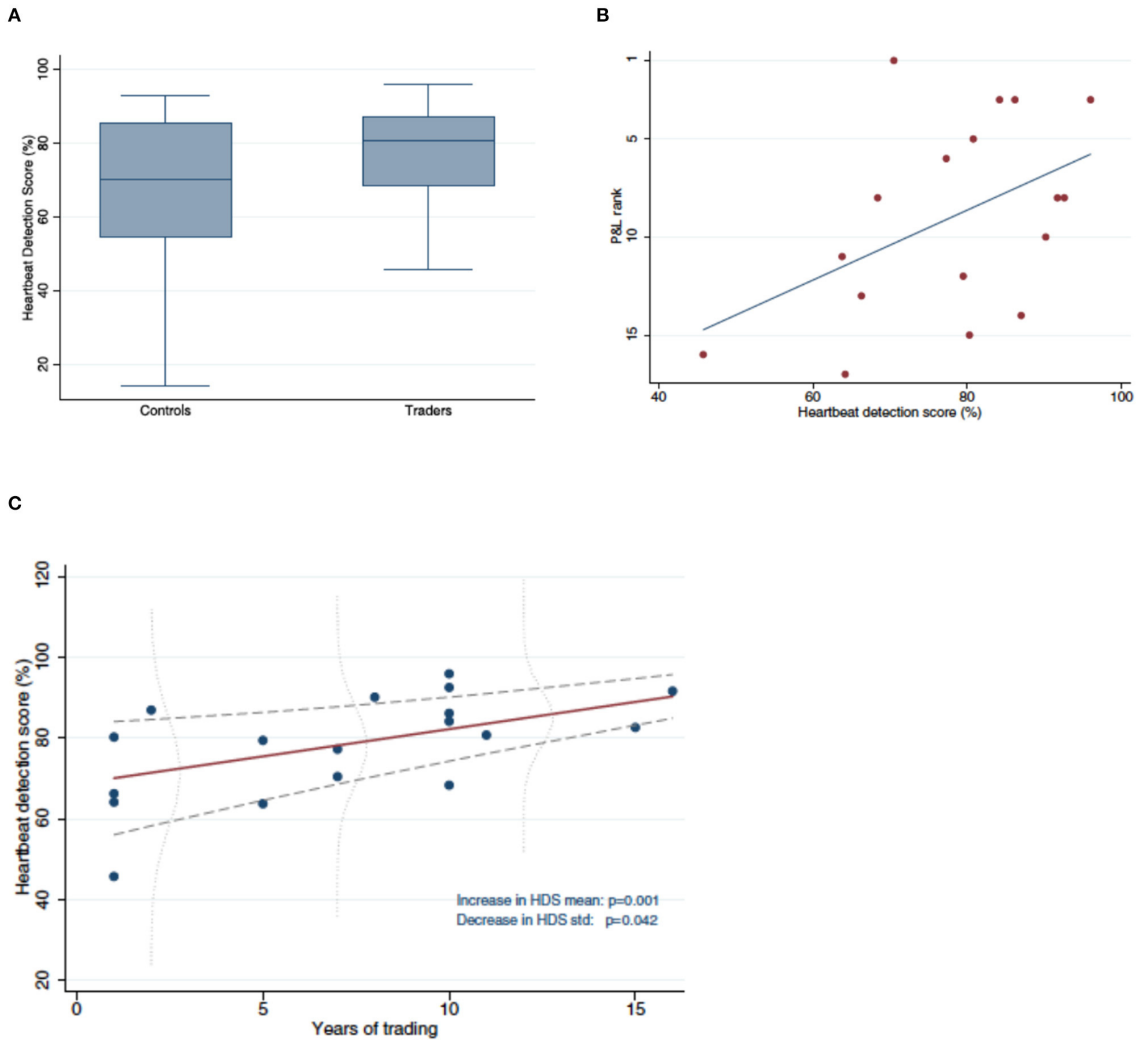
**(A)** Traders have significantly better heartbeat detection than Controls. Shown are boxplots of heartbeat detection scores for Traders (right) ad Controls (left). **(B)** More successful Traders have better heartbeat detection. Shown is the relation between heartbeat detection score and profit/loss (PandL) rank. **(C)** Traders with longer tenure have better heartbeat detection. Shown is the relation between years in trading and heartbeat detection score. Source: Kandasamy et al. (2016).

Our arguments will be based on an example. Recently, a group of neuroscientists and economists published an article in *Scientific Reports* that showed how intero-ceptive ability correlated with trading success on a London trading floor (Kandasamy et al., 2016). Specifically, it showed how professional traders' ability to sense their own heartbeat was better than that of the population at large and that this intero-ceptive ability correlated positively with their profit/loss performance, and with job tenure (Figure 1). These findings caused the *Financial Times* to conclude, rather sensationally, that “gut feeling” will eventually allow humans to beat robots (algorithmic traders)^1^.

Indeed, robots don't have hearts, so how can they ever acquire the intero-ceptive ability that traders appear to need to be successful? As we shall see, the answer to this question requires a deeper understanding of neurobiology. Is it really true that the human heart plays a role in human cognition that cannot be captured by a “rational” algorithm? Are emotions, of which heartbeat is one measure, orthogonal to rationality?

As we shall see, the heart in fact plays an integral role in rational decision-making. As do emotions in general. This is one of the main insights of recent studies of the neurobiology behind decision-making, i.e., *decision neuroscience*. The implications for economics and psychology, where emotions and reason are still widely believed to be antithetic, exceptions notwithstanding (Lerner et al., 2015), are profound.

The authors of the *Scientific Reports* study do not claim causality. As such, financial firms better not jump to the conclusion that they should hire traders on the basis of ability to sense their heartbeat.

Instead, the finding corroborated 15 years of research on the neurobiological foundations of human risk assessment and risk taking. Indeed, the finding really only makes sense if put into perspective against that research. It proved that this research can explain a strong, yet most puzzling link that exists between intero-ceptive ability and trading performance in financial markets. In fact, without the background research, one can quite reasonably question the validity of the finding, since it emerged in a sample of only 18 subjects (plus controls). The finding is only one piece in a chain of converging evidence on the role of one particular expression of emotional engagement, heartbeat, in successful financial decision-making.

To understand the link between the heart and decision-making in the context of risk and uncertainty, we first have to explore the links between the heart, the brain, and one key financial variable: volatility (or risk). What is to follow is a fascinating exploration of recent, seemingly unrelated findings, in financial decision-making and in neurobiology. Each finding is a piece in the puzzle that explains why traders who sense their heartbeat better make more money.

The goal of this article is not to provide a comprehensive review of the role of emotions in financial decision-making, and the neuroscience behind it. Instead, the article is meant to be a pedagogical tool for social scientists, and in particular finance scholars, to better comprehend, through a pointed example, how and why emotions form an integral part of reasoned decision-making. On the neuroscience side, for instance, the focus will be on a region called anterior insula, at the expense of other regions intimately connected to emotions, such as amygdala, anterior cingulate cortex, or even posterior parts of insula. The reason is simple: anterior insula has been associated with detection of heartbeat changes and conversion of those into anticipation of changes in the environment.

## Financial Risk Changes Correlate with Activation in Noradrenergic Neurons Which Drive Heartbeat Changes

**Figure 2:**
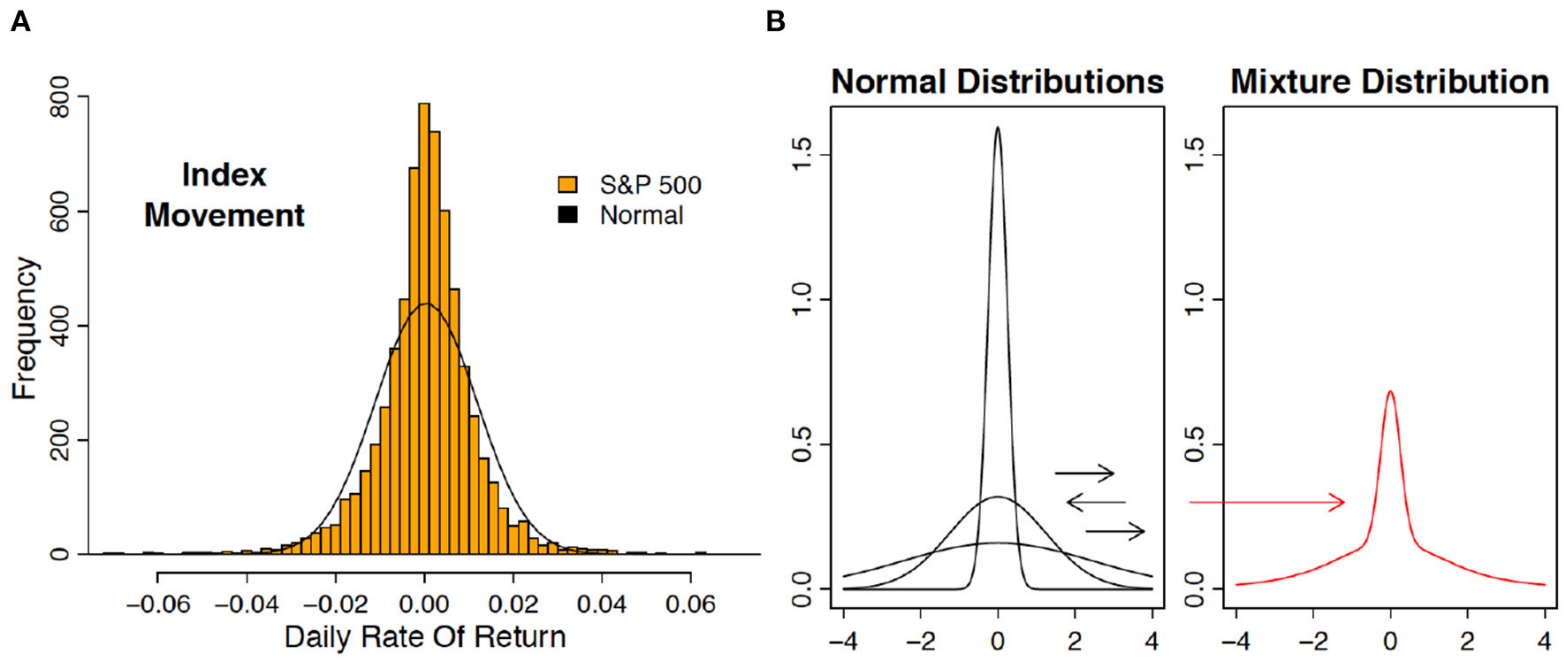
**(A)** The distribution of daily rate of return on the SandP 500 index is leptokurtic. Shown are the histogram of daily returns (1 June 1988–28 June 2013) and a Gaussian curve fit to the same data. If the Gaussian distribution had been correct, then a daily return over 4% in absolute value is expected to occur only once every 128 years. Over the 25 years displayed here, there were 41 such outliers. **(B)** Leptokurtosis can be obtained by shifting variance. Shown are three Gaussian curves with different variances (left). Repeated drawing by first choosing a variance and then drawing from the corresponding Gaussian curve (“mixing”) produces a leptokurtic distribution (right).

“So what are policymakers to do? First and foremost, reduce uncertainty. Do so by removing tail risks, and the perception of tail risks.” This statement, by the chief economist of the IMF^2^, summarizes what is unique about uncertainty generated in financial markets, namely, tail risk. Technically, one refers to leptokurtosis: outliers are far more prevalent than under the Gaussian distribution, and because small price changes are also more frequent, the outliers are immensely salient (see Figure 2A). It is thought that continuous changes in volatility generates this leptokurtosis (Figure 2B). GARCH (Generalized Autoregressive Conditional Heteroscedasticity) is one popular way to model volatility changes, and hence, leptokurtosis (Bai et al., 2003) (see also Box 1). There, outliers reveal increases in volatility.

Box 1 **GARCH and neural signaling in Anterior Insula.**

Neural signals in the Anterior Insula (AI; see **Box 4**) track errors in forecasting volatility. A popular way in financial econometrics to model changes in volatility is the Generalized Conditional Heteroscedasticity (GARCH) model (Bai et al., 2003). In its simplest form, this model assumes that return volatility σ_t_ over time t follows a first-order autoregressive process, driven by deviations from the mean (e_t_)^2^:

$$\begin{array}{l} {\left(\sigma_{\text{t}+1}\right)}^{2}=\phi {\left(\sigma_{\text{t}}\right)}^{2}+\psi {\left(e_{\text{t}}\right)}^{2}, \end{array}$$

where

$$\begin{array}{l} e_{\text{t}}=r_{\text{t}}-\mu , \end{array}$$

i.e., the shock e_t_ equals the deviation of the return on an asset over a period r_t_ from its expectation μ. The changes in volatility leads to “mixing,” which induces leptokurtosis in the return data. Preuschoff et al. (2008) discovered that Anterior Insula (AI) tracks risk prediction errors, defined as mistakes in volatility predictions. In the notation of the above GARCH model, risk prediction errors equal (e_t_)^2^ – (σ_t_)^2^, the difference between the realized squared deviation from the expectation and the expectation of this squared deviation. This “cool” mathematical quantity is tracked in AI, a key region of the “affective brain,” suggesting that mathematics is encoded through changes in emotions.

Outliers often revert, and automated high-frequency traders attempt to exploit those reversals, banking on the statistical regularity that reversals occur more frequently (Brogaard et al., 2018). That is, leptokurtosis often constitutes noise that can be taken advantage of, which is why it has been referred to as *leptokurtic noise* (D'Acremont and Bossaerts, 2016).

One can generate changes in volatility in a controlled setting, and study how the human brain reacts to it. Inserting electrodes into the brain is too invasive to be used in healthy humans and non-invasive methods like functional magnetic resonance imaging (fMRI) are too expensive and too elaborate to be used outside the lab (see Box 2). Fortunately, there are more easily accessible physiological measures that can serve as a proxy for the activity in particular brain regions. For instance, pupil dilation is known to reflect activation of the locus coeruleus (LC), a cluster of neurons in the brainstem, that mostly use the chemical norepinephrine (noradrenaline) to communicate with downstream neurons (see Box 3). The noradrenergic system is a key component of the attentional network in the brain. Noradrenaline in the brain increases arousal and alertness but also restlessness. As such maladaptive responses of this network are thought to be responsible for mental disorders such as Attention Deficit Hyperactivity Disorder (ADHD) and anxiety. Medications such as reboxetine (inhibitor) or guanfacine (agonist) regulate the noradrenergic system.

Box 2 **Non-invasive brain imaging techniques.**

There exist many techniques to “read” neural activation without physically going into the brain. One of the most popular (but expensive) is *functional Magnetic Resonance Imaging* (fMRI), which indirectly, and with a delay, picks up neural activation by tracking oxygen-rich blood that flows to clusters of neurons that have “fired.” The fMRI scanner creates a very strong magnetic field, which it disturbs, resonating with oxygen atoms. By recording the resonance, the scanner can identify time and location of the oxygen.

There are many other ways to detect neural activation, such as EEG (Electro-Encephalogram). Recently neuroscientists have come to realize that there are effective and simple ways to track firing by specific clusters of neurons. One cluster is LC, where noradrenergic neurons are located. Firing in that cluster has an effect on *pupil dilation*, and as such, pupil dilations constitute a “mirror” of LC activation, provided of course there are no other reasons for the pupil dilation, such as changes in luminosity (Joshi et al., 2016).

Box 3 **Neurons and neurotransmitters.**

Information processing in the brain is done by a certain type of cell called *neuron*. The neuron receives signals from upstream neurons through many of its *dendrites* (“roots”) and collects those signals in the form of electrically charged molecules (ions) into its cell body. Together, cell bodies form the “gray matter” of the brain. If the information carried by those ions is sufficiently strong, the neuron “fires” by sending a charge through its *axon* to downstream neurons it has connected with. As such, neuronal signals are basically binary: to fire or not; like the transistors in a modern electronic computer. The neuron does not physically connect to its downstream neurons. Instead, there is a *synaptic cleft* into which the neuron, if it fires, releases chemicals called *neurotransmitters*, which the downstream neurons will pick up – unless inhibited somehow, e.g., through drugs that neutralize receptors on the downstream neuron. The brain uses many types of neurotransmitters. Some of them don't merely “send information,” but rather modulate information transmission, enhancing or reducing the impact of neural signals. E.g., dopamine, serotonin, norepinephrine (or noradrenaline), acetylcholine. Neuropharmacology in general attempts to affect information transmission in the brain by targeting specific neurotransmitters, directly (e.g., inhibiting their re-uptake out of the synaptic cleft; e.g., Prozac), or indirectly (increasing the sensitivity of the receptors of the downstream neurons; e.g., reboxitine).

Preuschoff and collaborators exploited this link between pupil dilation and noradrenaline to study the effect of (changes in) volatility on neural activity. They had participants play a simple card game. To suppress changes in pupil dilation due to changes in luminosity (the well-known pupillary light reflex), all stimuli were presented aurally rather than visually (Preuschoff et al., 2011). Throughout the card game, volatility (measured as standard deviation of expected payoff) changed constantly. The researchers found that pupil dilations were strongly correlated with mistakes in predicting volatility. That is, pupil dilations “measured” risk prediction errors – the driving term in the popular GARCH processes. Remember that the task was entirely auditory and these changes can therefore not be explained by changes of luminosity in the surroundings. Instead the changes in pupil dilation likely reflected changes in neural activity – most likely the activity of noradrenergic neurons in LC or its afferent (upstream) and efferent (downstream) brain circuitry.

Noradrenergic neurons have projections into many parts of the brain, such as the pre-frontal cortex and the visual cortex. In addition, some projections reach the heart without passing through the brain, causing heartbeat modulations. In this sense, changes in heartbeat too could be conceived as indirect measurement of noradrenergic activity, just like pupil dilation.

Despite their far-reaching projections, noradrenergic neurons are unlikely to be the source of complex behaviors or emotions on their own, since the regions whence they originate are tiny and there are too few of these neurons. To understand complex behaviors we have to look to the cortex.

The anterior insula (AI) is a cortical structure in humans (as well as in primates and many other species such as dolphins and whales) which is thought to be responsible for translating emotions – which we define to be bodily reactions as measured in psychophysiology, such as heartbeat, transpiration, blood pressure, etc. – into feelings. Indeed, through AI we become aware of our emotions (Craig, 2014). Not surprisingly, AI activates in reaction to pain and disgust, but also to empathy, effectively “simulating” the emotional reactions of others (Singer et al., 2004) (see also Box 4).

Box 4 **Anterior Insula.**

Anterior Insula (AI) is a widely connected cortical structure generally understood to integrate information from various sources, thereby providing a meeting point for sensory, autonomous, affective and cognitive inputs into decision-making. The integrative role of AI explains why activation can simultaneously correlate with bodily phenomena such as pain or disgust and reflect complex mathematical quantities such as risk prediction errors (see Box 4). AI also plays a crucial role in self-awareness, enhanced during ecstatic epileptic seizures, which are thought to be caused by a brain network centered around AI. Together with Anterior Cingulate Cortex, the human AI contains peculiar neurons, von Economo neurons, distinguished by their simple dendritic structure (Butti et al., 2013). It is thought that these neurons allow for fast adaptation in an uncertain environment that continuously generates novel circumstances, bypassing the intricate neural network structure of regular, pyramidal neurons. Selective activation of AI under leptokurtic noise, a type of risk that is associated with modern financial markets, but not the traditional, natural environment humans had to navigate, could be one example of how AI specializes in dealing with challenging novel situations.

**Figure 3:**
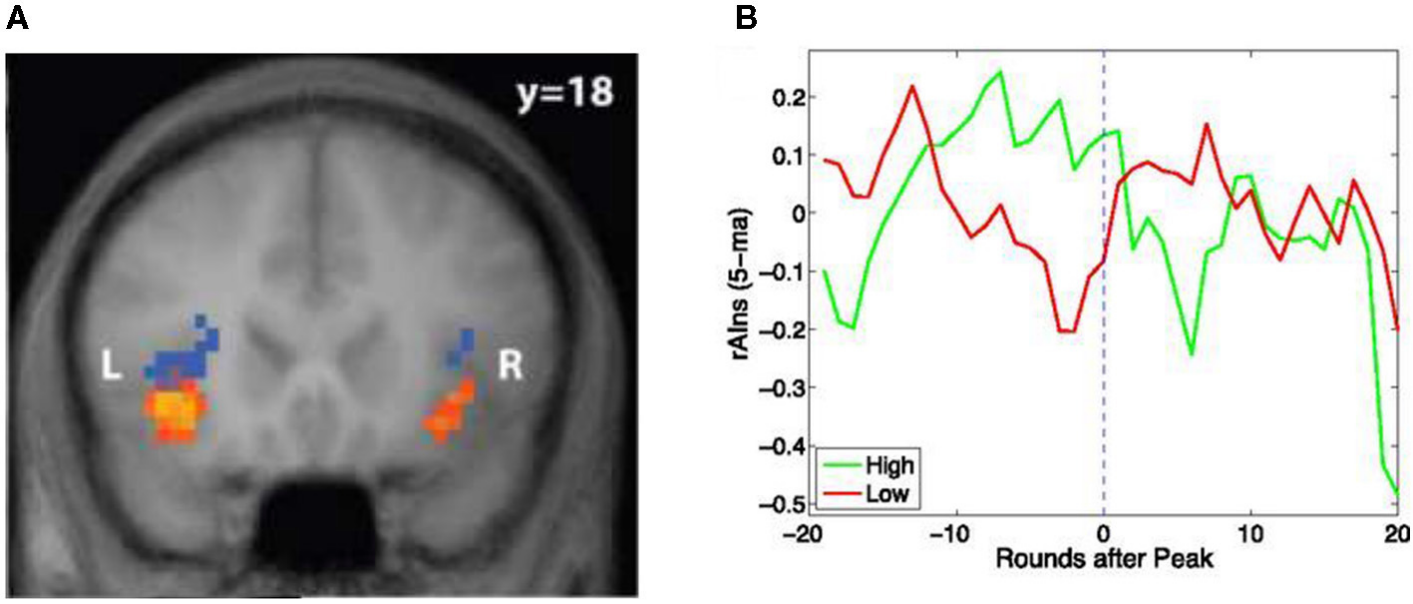
Blue regions predict the size of rewards or losses, i.e., the risk. Orange regions track the prediction mistake. Mistakes happen when the size of the actual reward or loss is much bigger than anticipated, i.e., upon an outlier. The regions form part of the Anterior Insula (AI). Based on functional magnetic resonance imaging of brain activation during a card game where the predicted size of reward or losses changes constantly. **(B)** Activation in the region within right AI (rAIns) predicts which traders correctly anticipate the bursting of a financial bubble. Shown is the evolution of activation in the red region next to “R” in **(A)** before and after the trading round when the bubble peaked. Red line is for participants who anticipated that the bubble would have lasted longer. Green line is for participants who correctly anticipated the crash. Sources: **(A)** Preuschoff et al. (2008) and **(B)** Smith et al. (2014).

With hindsight, it is therefore not surprising that AI is involved in risk tracking as well. In a visual version of the same card game used in the pupil dilation study, we discovered that AI activation correlated with risk anticipation *as well as risk prediction errors* (Preuschoff et al., 2008) (see Figure 3A). The study was the first to discover cool, rational mathematical signals in a brain structure that had been associated with emotions, feelings, and awareness, phenomena that were thought to defy formal analysis. It was also one of the key pieces of mounting evidence that emotions were an integral part of rational calculations, thereby casting serious doubt on the widespread belief that the affection (emotions) and cognition (reason) were antagonistic (We will return to this antagonism later.).

The above findings served to finally make sense of a much earlier study. That study was the first to monitor, on the job, the psychophysiology of professional traders, in to contrast to amateurs. Way before the link between outliers, LC and AI was established, it was indeed shown that professional traders reacted emotionally in a very narrow way to participation in financial markets; significant correlation only emerged between changes in heartbeat and *changes in market volatility* (Lo and Repin, 2002).

More evidence has been piling up in recent years. For instance, Payzan-LeNestour et al. (2013) showed direct evidence between LC activation and outliers in a study where outliers were generated differently, namely, through shifts in the mean of the payoff distribution; Nassar et al. (2012) showed that such outliers also drove pupil dilation; AI was found to be engaged in differentiating between frequent outliers that reverted (leptokurtic noise) and outliers that did not (D'Acremont and Bossaerts, 2016).

AI, through its interaction with the heart, thus emerged as the crucial brain structure involved in tracking the very essence of financial risks, namely, leptokurtosis. It turns out that something important was already known about the link between heartbeat changes and AI. Let us now discuss this particular neurophysiological interaction.

## Size of Anterior Insula Correlates with Heartbeat Detection Accuracy

In 2004, one of the authors of the study of the intero-ceptive capability of professional traders, collaborated on a study of the link between one's ability to sense heartbeat and the size of the AI. The results were reported in Critchley et al. (2004). There, the size of AI was found to correlate with differences in accuracy in determining one's heartbeat.

This study provides the missing link between the aforementioned studies on the role of AI in tracking financial risks and the link between heartbeat sensing and trader performance. To put it all together: the better the connectivity between heart and AI, the better one's senses are “in tune” with changes in financial risks, and hence, the better trader one becomes.

Again, there is no causality meant. It is not clear whether the increased size of AI in better “heartbeat trackers” is its cause or its consequence. The evidence merely points to a strong link in the system financial risk/LC/heartbeat/AI and trading performance. Without the rich neurobiological evidence, however, the correlation between ability to sense heartbeat and trading success could as well have been a fluke. The evidence from the earlier neurobiology studies is consistent with, and provides foundation to, this extraordinary discovery, supporting its credibility.

As the evidence converged, researchers were left with the question of whether any of these physiological signals could be used to predict or drive behavior in complex financial markets. They could, as we explain next.

## More on Anterior Insula: How to Get Out of a Bubble in Time

AI had been taking a central position in studies of financial decision-making as its activation had been consistently linked to risk and outliers in controlled experiments. As such, it appeared to be a prime candidate for tracking and driving behavior in more complex settings, such as trade in financial markets.

In one experiment, participants traded in an online market setting that is known to generate bubbles – prices that are far above fundamental values in the sense that they are higher than even the sum total of dividends that will ever be paid until the end of the experiment. Some traders participated from inside a scanner. This meant that they could submit orders and trade while their brain activity was being recorded using functional magnetic resonance imaging (fMRI). A number of participants “rode” the bubble: they bought when the security was clearly over-priced, presumably hoping that they would be able to sell in time, before the bubble burst.

Remarkably, this study, Smith et al. (2014), showed that brain activation in AI could be used to predict who would get out in time. They tracked brain activation in the same part of AI where Preuschoff et al. (2008) discovered neural signals correlating with risk prediction errors (see above). Participants with significantly higher AI activation during emergence of the bubble managed to get out in time, thus performing much better than those with lower AI activation (see Figure 3B).

Neuroscientists associate AI with emotions, feelings and self-awareness. The crucial role that AI appears to be playing in successfully dealing with financial risks may therefore lead one to conjecture that emotions are an integral part of sound financial decision-making. As it turns out, this link between emotions and financial decision-making had already been made in the 90s, by two neurologists.

## Emotions are a Necessary Condition for Sound Financial Decision-Making

In the 90s, neurologists noticed that patients with certain lesions in the orbitofrontal cortex (OFC) appeared to make worse financial decisions after they acquired these lesions. OFC is large region that borders on AI, and brain lesions tend to be diffuse, which means that if they affected OFC, they were likely to impact borderline regions as well. The neurologists set out to test their patients' ability to choose rationally by means of controlled experiments.

The task they gave their patients, the *Iowa Gambling Task*, is effectively a four-armed bandit problem in the form of a card game. Unbeknown to the participants, two arms dominated, in the sense that they generated payoffs that were better both in terms of expected payoff (positive rather than negative) and in terms of risk (variance and range of payoffs). Against healthy controls, patients continued to choose the bad arms long after it should have become clear that they were dominated, and even though they expressed awareness of their higher risks and lower returns.

Significantly, the neurologists discovered that their patients had no emotional anticipation of the risks they were taking (Bechara et al., 1997). In particular, unlike healthy controls, they did not exhibit anticipatory anxiety, in the form of transpiration (measured by changes in skin conductance) when choosing the inferior, high-risk arms.

This amounted to the first evidence, in the context of finance, that the traditional picture of a tension between affection (emotions) and cognition (reason) was wrong. Yet the view that emotions stand in the way of rational decision-making is still widely promoted in economics and psychology. To counter this, in an article in a 2005 issue of *Games and Economic Behavior*, the aforementioned neurologists emphasized that “[e]merging neuroscience evidence suggest that sound and rational decision-making, in fact, depends on prior accurate emotional processing” (Bechara and Damasio, 2005).

The importance of emotions in reasoned decision-making is a recurring theme in decision neuroscience. In 2017, neuroscientists discovered that higher susceptibility to losses than to gains (“loss aversion”) is *not* the result of increased activation of emotional neural circuits, but of reduced overall task engagement. In one sense, they actually found the contrary: *choices became more rational upon increased engagement of both emotional and affective brain circuitries* (Li et al., 2017).

## In Summary

At first, the findings from the *Scientific Reports* article of a correlation between the ability to track reliably one's heartbeat and performance of professional traders in financial markets seem odd. Without further evidence one might have been tempted to dismiss them as spurious, certainly in view of the small sample size, and unlikely to be replicable. Yet, not only did further evidence exist, it explained why the correlation emerged.

Emotions are an integral part of rational decision-making. As such, a trader who cannot sense own heartbeat is at risk to underperform, and not to last long on the job. This does not mean that emotions are good *per se*. In 2012, Fenton-O'Creevy et al. (2012) provided a qualitative investigation of how antecedent-focused heartrate regulation improved trading performance, but response-focused regulation did not. Recently, Bossaerts et al. (2020) confirmed the finding quantitatively, using the same experimental paradigm as in Smith et al. (2014).

In view of these and other findings we argue that the claim that emotions “have not been incorporated into the economic theory of decision-making under uncertainty” [p. 55, Caplin and Leahy (2001)] is no longer tenable: if emotions contribute to maximizing utility, then somehow they are an integral part of rational decision-making. Where exactly they show up in the mathematics is yet to be determined in detail, and will require collaboration between economists and neuroscientists, in the tradition of neuroeconomics.

## Data Availability Statement

Publicly available datasets were analyzed in this study. This data can be found here: https://academic.oup.com/cercor/article/26/4/1818/2367610#supplementary-data.

## Author Contributions

The author confirms being the sole contributor of this work and has approved it for publication.

## Conflict of Interest

The author declares that the research was conducted in the absence of any commercial or financial relationships that could be construed as a potential conflict of interest.

## Footnotes

^1^“Man v machine: ‘Gut feelings’ key to financial trading success.” *Financial Times* 19 Sep 2016.

^2^Olivier Blanchard, chief economist, IMF, *The Economist* 31 January 2009.
